# Tuning the Photoluminescence of Graphene Quantum Dots by Photochemical Doping with Nitrogen

**DOI:** 10.3390/ma10111328

**Published:** 2017-11-20

**Authors:** Xiaofen Xu, Fuhua Gao, Xiaohua Bai, Fuchi Liu, Wenjie Kong, Ming Li

**Affiliations:** 1 College of Physics and Technology, Guangxi Normal University, Guilin 541004, China; xiaofenxu111@163.com (X.X.); 15078369629@163.com (F.G.); 18593231070@163.com (X.B.); 2 College of Science, Guilin University of Technology, Guilin 541004, China; liming928@163.com

**Keywords:** graphene quantum dots, nitrogen-doped graphene quantum dots, photochemical doping, photoluminescence, blue-shift

## Abstract

Nitrogen-doped graphene quantum dots (NGQDs) were synthesized by irradiating graphene quantum dots (GQDs) in an NH_3_ atmosphere. The photoluminescence (PL) properties of the GQDs and the NGQDs samples were investigated. Compared with GQDs, a clear PL blue-shift of NGQDs could be achieved by regulating the irradiating time. The NGQDs obtained by irradiation of GQDs for 70 min had a high N content of 15.34 at % and a PL blue-shift of about 47 nm. This may be due to the fact that photochemical doping of GQDs with nitrogen can significantly enhance the contents of pyridine-like nitrogen, and also effectively decrease the contents of oxygen functional groups of NGQDs, thus leading to the observed obvious PL blue-shift.

## 1. Introduction

In recent years, graphene quantum dots (GQDs) have emerged as promising optical materials with prospective applications in bio-imaging, fluorescence, and cancer diagnosis and treatment due to their unique physical, chemical and biological properties [1,2,3,4]. However, there are still some issues that need to be improved such as the narrow spectral coverage and the lack of tailor-made control of their optical properties [5]. It has been demonstrated that doping the GQDs with nitrogen atoms can change their physical and chemical properties, and significantly tailor their photoluminescence (PL) [6,7,8]. To this end, N-doped GQDs (NGQDs) have been synthesized by many methods described in References [8,9,10]. For example, Nateghi et al. [6] developed a microwave-assisted hydrothermal method to synthesize blue-luminescent NGQDs from glucose and ammonia as carbon and nitrogen sources, respectively. Yang et al. [11] reported a bottom-up method to synthesize yellow-luminescent NGQDs through the pyrolysis of 1,3,5-triamino-2,4,6-trinitrobenzene at 750 °C under a nitrogen atmosphere. Jeon et al. [1] presented an approach for preparing the amine group-functionalized GQDs exhibit a redshift of about 30 nm compared to that of the unfunctionalized GQDs by reacting graphene oxides with diamine terminated polyethylene glycol.

Obviously, NGQDs prepared by different means show diverse PL properties, and the PL shifting of the GQDs through chemical doping or functionalization requires further study. In addition, the methods discussed above for synthesizing NGQDs are limited by the availability of special equipment, special chemical reagents, high temperature, or long production phases. Therefore, a simple approach to prepare NGQDs is desirable. 

Compared with conventional doping means, the photochemical doping method is low-temperature, rapid, green, and easy to control via irradiation [12]. It has been widely used in preparing doped graphene oxide, and reduced GQDs [13,14]. For applications in cell labelling, protein trafficking, DNA detection, etc., water-soluble and luminescent quantum dots are highly desired [15]. In addition, for practical applications in light emitting devices and short-wavelength diode lasers, a wide bandgap or short-wavelength semiconductor is highly desirable [16]. It has been demonstrated that oxygen-rich and water-soluble nitrogen doped graphene oxide can be obtained by a photochemical doping method [14]. Here, we report on the PL shift of GQDs through N-doping through a photochemical method. The results showed that when the GQDs were irradiated for 70 min, the N content of NGQDs obtained could reach up to 15.34 at %. More interesting, N-doping resulted in tunable PL of GQDs, and the maximal PL blue-shift of GQDs could reach 47 nm.

## 2. Experimental Section

### 2.1. Materials

All chemicals were used as received. Vulcan XC-72 carbon black was purchased from Cabot Corporation (Boston, MA, USA), nitric acid (15 mol/L) was bought from Alfa Aesar (Shanghai, China), and NH_3_ (99.99%) was bought from Guoxin Corporation (Nanning, China).

### 2.2. Instrumentation

Transmission electron microscopy (TEM) was performed on JEM-2100F (JEOL, Tokyo, Japan). IR spectra were recorded on a Fourier transform infrared spectroscopy (FT-IR) spectrometer (Perkin-Elmer, Waltham, MA, USA). X-ray photoemission spectroscopy (XPS) measurements were performed on ESCALAB 250Xi (Thermo Fisher Scientific, Waltham, MA, USA) using Al Kα radiation. The PL spectra were measured at ambient conditions by a spectrofluorophotometer (RF-5301PC, Shimadzu, Kyoto, Japan) using a Xe lamp as the light source.

### 2.3. Preparation of N-Doped GQDs (NGQDs)

As reported previously, the GQDs were synthesized with Vulcan XC-72 carbon black by means of chemical reflux [17]. The NGQDs were prepared by a similar method of the synthesis of nitrogen-doped graphene oxide through a photochemical route [14]. Briefly, the GQDs were put in a quartz boat, and were then placed inside the quartz tube. With the quartz tube located in two brackets, the GQDs were irradiated for a target time with light from a 500 W high-pressure Hg lamp in NH_3_ (~99.99%) atmosphere at a rate about 60 mL min^−1^. After cooling the quartz tube to room temperature, the NGQDs samples (NGQDs-10, NGQDs-30, NGQDs-50, NGQDs-70, NGQDs-90, numeric numbers denote the irradiation time) were obtained.

## 3. Results and Discussion

### 3.1. Transmission Electron Microscopy (TEM) Images of the Graphene Quantum Dots (GQDs) and N-Doped GQDs (NGQDs)

**Figure 1:**
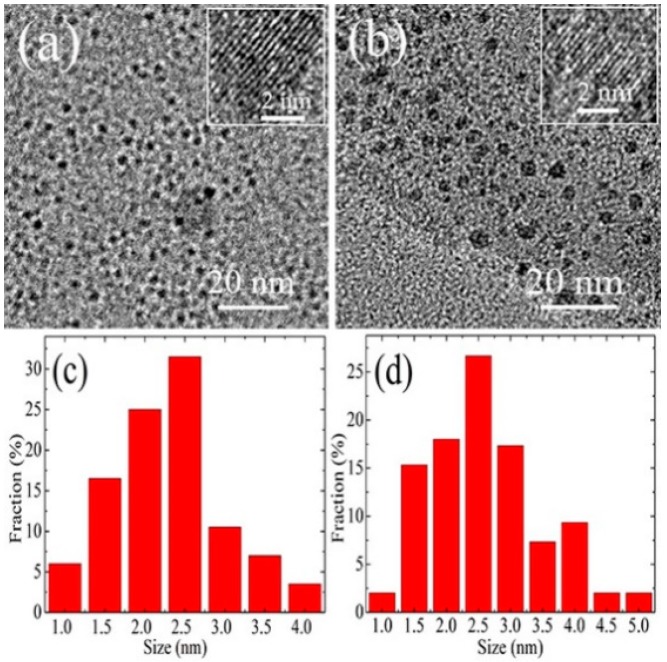
Transmission electron microscopy (TEM) images of (**a**) graphene quantum dots (GQDs) and (**b**) NGQDs-70 (numeric number denotes the irradiation time). Diameter distributions of (**c**) GQDs and (**d**) NGQDs-70.

Figure 1a–d shows the typical TEM images and diameter distributions of GQDs and NGQDs-70. It was found that the GQDs and NGQDs-70 were relatively uniform with diameters of about 1–4 nm and 1–5 nm, respectively. According to the calculations, one found that the average diameters of the GQDs and NGQDs-70 are approximately 2.3 and 2.6 nm, respectively (Figure 1c,d); however, no noticeable change was observed in their sizes. The insets of Figure 1a,b show the high resolution transmission electron microscopy (HR-TEM) images of GQDs and NGQDs-70, which demonstrate that the GQDs and NGQDs-70 have clear discernible lattice structures.

### 3.2. Raman, and Fourier Transform Infrared Spectroscopy (FT-IR) Spectra of GQDs and NGQDs

**Figure 2:**
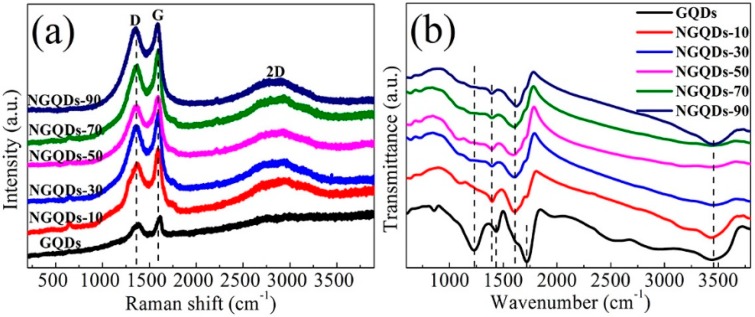
(**a**) Raman spectra; and (**b**) Fourier transform infrared spectroscopy (FT-IR) spectra of the GQD and NGQD samples.

Figure 2a shows the Raman spectra of the GQD and NGQD samples obtained at different irradiation times. One can see that there are two prominent peaks of the D and G band at 1386 and 1613 cm^−1^ of the GQDs, respectively. It is known that the D band is a disordered band arising from the disorder in the sp^2^ hybridized carbon, while the G band corresponds to the first-order scattering of the stretching vibration mode *E*_2*g*_ observed for the sp^2^ carbon domains [18]. The broad band centered at approximately 2750 cm^−1^ corresponds to the 2D band, which arises from the relaxation in selection rules caused by phonon scattering at the boundaries and defects in the GQDs [19]. Compared with the pristine GQDs, the Raman spectra of the NGQDs showed some peak shift. The D peaks were blue-shifted by approximately 17–28, and the G peaks were blue-shifted by 11–24 cm^−1^. Generally, the intensity ratio of the D band (*I_D_*) to G band (*I_G_*) (*I_D_/I_G_*) is used to estimate the disorder of carbon based nanomaterials [20]. It can be seen that all the NGQD samples showed higher *I_D_/I_G_* (0.87–0.98) than the GQDs (0.86). Moreover, similar to the N-doped graphitic materials reported [21,22], the shift of the G band was due to the structural distortion of the NGQDs caused by the different bond distances of C-C and C-N. Thus, the larger *I_D_/I_G_* and blue-shift of the G band observed for the NGQD samples suggested N doping in the NGQDs.

FT-IR analysis (Figure 2b) was used to characterize and ascertain the surface functional groups present in the GQD and NGQD samples. The FT-IR spectra showed broad absorption bands in the GQDs and NGQDs from 3000–3500 cm^−1^, which are characteristic absorption bands for -OH stretching vibrations [23,24]. The absorption bands at around 1718, and 1434 cm^−1^ corresponded to the stretching vibrations of C=O, -COOH, respectively. The weak peaks at around 1617, and 1394 cm^−1^ were due to the C=N, C-N stretching and bending, respectively. The absorption peaks at 1220–1250 cm^−1^ were attributed to C-OH, C-C stretching, and -OH bending vibrations [25]. After reduction and functionalization with NH_3_, the stretching frequency (1718 cm^−1^) corresponding to C=O shifted to 1617 cm^−1^ due to the formation of amide bonds [26]. 

### 3.3. X-ray Photoemission Spectroscopy (XPS) of GQDs and NGQDs

To detect the nitrogen content and the bonding environment of the C and N species of the NGQD samples, X-ray photoemission spectroscopy (XPS) was employed. As shown in Table 1, the oxygen and N contents of the samples were defined as 100 O/C at % and 100 N/C at %, respectively. It was found that the GQDs had a high oxygen content of 58.88 at %, implying that they were oxygen-rich. In contrast, NGQDs-30 had a relatively low O content of 18.80 at %, but the highest N content of 22.56 at %. This suggested that after irradiation for 30 min without extra heating, GQDs were reduced and doped with high-content N. Furthermore, it has been confirmed that oxygen groups in graphene oxide facilitate reactions with NH_3_ and C-N bond formation [27]; namely, the enriched reactive oxygen functional groups in GQDs provide ample covalent bonding sites for chemical functionalization, and facilitate reactions with NH_3_ and C-N bond formation. Furthermore, one can see that (i) only being irradiated for 10 min, NGQDs-10 had a high N content of approximately 18.08 at %; and (ii) with an increase in irradiation time, the N content of the NGQDs samples remained relatively steady, and in the range of 15.34–22.56 at % (Table 1). However, it was found that enough irradiation time (>10 min) was necessary for the effective decomposition of carboxyl groups that facilitate the low O content of the NGQD samples. For example, despite a high N-A content, NGQDs-10 had a relatively high O content (approximately 29.17 at %). With an increase of irradiating time, the O content of the NGQD samples remained relatively steady.

**Figure 3:**
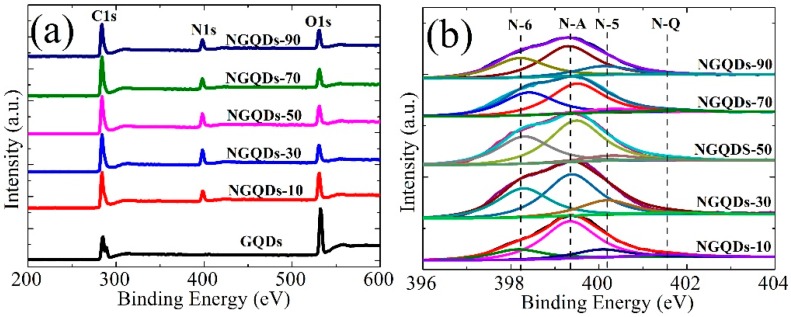
X-ray photoemission spectroscopy (XPS) spectra of the GQD and NGQD samples. (**a**) Wide scan urvey; (**b**) Fine-scanned N1s XPS spectra of the NGQD samples obtained at different irradiation times.

Figure 3 shows the XPS spectra for the GQD and NGQD samples. As shown in Figure 3a, one can see that (i) the NGQD samples showed clear N signal peak at approximately 400 eV; and (ii) the intensities of the O1s peak of the NGQD samples were much lower than those of the GQDs; however, (iii) the intensities of the C1s peak of NGQD samples were much higher than those of the GQDs. To obtain information on the incorporation of nitrogen into carbon, the N1s spectra of the NGQDs samples were fine-scanned and each peak was deconvoluted into four subpeaks (Figure 3b). The four peaks at 398.3, 399.4, 400.2, and 401.7 eV, were assigned to pyridine-like nitrogen (N-6), amino-like nitrogen (N-A), pyrrolic-like nitrogen (N-5), and graphitic-like nitrogen (N-Q), respectively [17,28,29,30,31]. Clearly, the fitted curves were more aligned with the experimental curve with a four-component fitting, and all the fitted peaks remained in a similar position for all the NGQD samples, which suggested the validity of the four-component assignments. Based on the XPS results, the content of the specific N types of the NGQD samples was obtained at different irradiating times are quantitatively shown in Table 1. One can see that with increasing irradiating time, (i) the contents of both N-A and N-5 displayed an up-and-down trend, the N-A reached the maximum of 11.62 at % for NGQDs-10, and N-5 reached the maximum of 3.73 at % for NGQDs-30; and (ii) the contents of N-Q remained relatively steady. Typically, when the irradiation time was over 10 min, all the NGQDs had high contents of pyridine-like nitrogen. Moreover, all the produced NGQDs contained large amounts of amino-groups (Figure 2b and Figure 3b, and Table 1). It was confirmed that the amino-groups may be preponderantly located in the edges of graphene layers [29], which may lead to the somewhat disordered structure in the NGQDs, and induced a higher *I_D_/I_G_* of NGQDs in the Raman spectra.

### 3.4. Photoluminescence (PL) Properties of the NGQDs 

**Figure 4:**
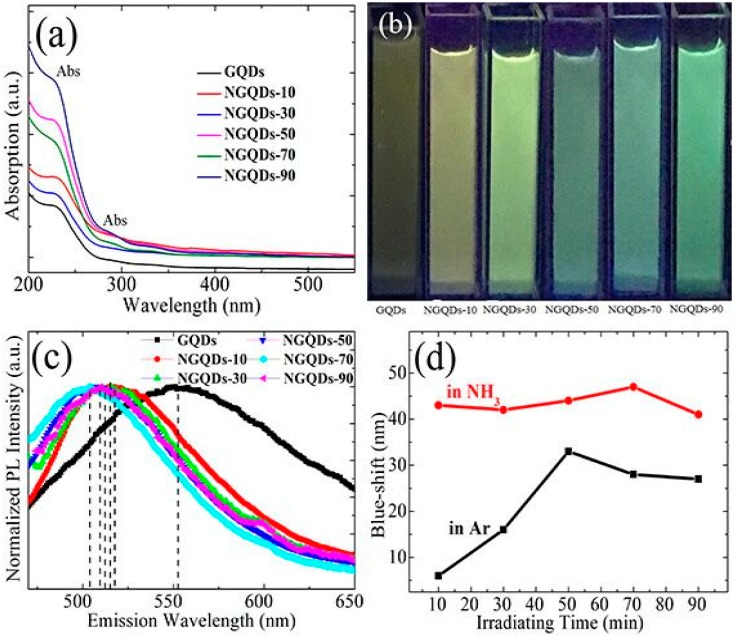
(**a**) UV-vis absorption spectra of the GQDs and NGQDs; (**b**) Optical photograph of the GQD and NGQD samples under irradiation from a UV lamp with a wavelength of 365 nm; (**c**) Normalized photoluminescence (PL) spectra of the GQD and NGQD samples excited at 400 nm; (**d**) The dependence of the PL blue-shift of the NGQDs and rGQDs compared to the GQDs on irradiating time.

To explore the optical properties of the NGQD samples, photoluminescence (PL) and UV-vis absorption spectra were performed. The PL result of the GQDs was also measured as a comparison. The UV-vis absorption spectrum of the GQDs showed an absorption band at approximately 240 nm (Figure 4a). However, an absorption band at circa (ca.)240 nm and another absorption band at ca. 300 nm could be seen in the UV-vis absorption spectra of the NGQDs. Figure 4c shows the normalized PL intensity of the GQD and NGQD samples with an excitation wavelength of 400 nm. It was found that when compared to that of the GQDs, the PL emission peaks of the NGQDs varied with the irradiation time in the range of 504–510 nm and retained a sharp full-width at half-maximum (FWHM) as narrow as 96–110 nm. In other words, the PL of all the NGQD samples exhibited a clear blue-shift. As shown in Figure 4b, the aqueous suspensions of NGQDs exhibited bright colorful luminescence varying from pale yellow to blue-green under irradiation from a UV lamp with a wavelength of 365 nm. 

It is well-known that a reduction reaction always accompanies the doping process. Thus, to approximately clarify the role of oxygen functional groups in the PL blue-shift of the NGQD samples, the PL spectra of the reduced GQD (rGQD) samples obtained from the GQDs irradiated in Ar were also measured. It was found that all rGQD samples showed lower O contents (ca. 35.63–49.43 at %) and PL blue-shift when compared to the GQDs (Figure S4). The PL blue-shift level of the NGQDs and rGQDs was defined as (P_GQDs_-P_NGQDs_) and (P_GQDs_-P_rGQDs_), respectively. Herein, P_GQDs_ is the PL emission peak position of the GQDs, and P_NGQDs_ and P_rGQDs_ are the PL emission peak position of the NGQDs and rGQDs, respectively. As shown in Figure 4d, the red and black symbol curve shows the dependence of the PL blue-shift of the NGQDs and rGQDs on irradiation time, respectively. Below 70 min, the PL blue-shift level of the NGQDs displayed an upward trend with increasing irradiating time, and reached the maximum blue-shift of approximately 47 nm for NGQDs-70. Above 70 min, as the irradiation time increased, the PL blue-shift level of the NGQDs decreased. It could be seen that rGQDs-50 presented the maximum PL blue-shift of approximately 33 nm, much lower than those of the NGQDs samples. Obviously, all the PL blue-shift level of the NGQDs are much higher than those of rGQDs. In other words, the photochemical doping with nitrogen plays more important role than only photochemical reduction in the blue-shift of GQDs.

Interestingly, the observed PL blue-shift phenomenon of the NGQDs was very different from the results reported in Reference [1]. Due to the charge transfers between the functional groups and GQDs, the PL emission of the GQDs functionalized with amine groups exhibited a red-shift when compared to that of the unfunctionalized GQDs [1]. Considering that the N-A dominated the N-doping of the NGQDs, an opposite PL shift was observed, which suggested that the origin of the PL blue-shift of the NGQDs was complex. 

It has been demonstrated that the band gap of the GQDs increased as the size of the GQDs decreased [32,33]. For example, Peng et al. [34] reported that the PL emission of the GQDs was blue-shifted when their size decreased due to the increased band gap of the GQDs. However, this behavior was inconsistent with our experimental phenomenon. Our results demonstrated that doping the GQDs with nitrogen did not significantly alter their size (Figure 1a–d). Therefore, we estimated that the PL blue-shift of the NGQDs was not primarily influenced by their size due to the quantum confinement effect.

It has also been reported that N doping in pyridinic sites induced a blue-shift in the PL maximum due to the high electronic affinity of the nitrogen atom [10]. In view of the fact that NGQDs have high contents of pyridine-like nitrogen (N-6) (Table 1 and Figure 3), pyridine-like nitrogen doping may play an important role on the observed PL blue-shift of the NGQDs. In addition, it is known that as the content of oxygen functional groups of GQDs decreases, an obvious PL blue-shift of the GQDs can be seen [35]. It has been confirmed that xenon lamp radiation can cause chlorine molecules to split into highly reactive chlorine radicals, which presumably combine with the graphene through a free radical addition reaction [36]. In this study, the NGQDs were prepared by radiating the GQD samples with a similar mercury lamp under an ammonia atmosphere. Similarly, ammonia molecules were split into highly reactive ammonia radicals, which combined with GQDs through a free radical addition reaction. Considering that the NGQD samples had much lower contents of oxygen than rGQDs, it was suggested that highly reactive ammonia radicals facilitate the decrease of the content of oxygen functional groups of GQDs more than argon under mercury lamp radiation, and that the low contents of oxygen functional groups of the NGQDs may also induce the PL blue-shift of the GQDs. Therefore, it demonstrated further that doping GQDs with nitrogen through photochemical means could effectively enhance the contents of pyridine-like nitrogen, and also effectively decrease the contents of the oxygen functional groups of NGQDs, which may be mainly responsible for the observed obvious PL blue-shift of the NGQDs. 

From the results of the TEM, Raman, FT-IR, XPS, and PL analysis, one can see that the NGQDs had high contents of pyridine-like and amino-like nitrogen, but had low contents of oxygen. However, doping did not noticeably change the sizes of the NGQDs, that is, the PL blue-shift of the NGQDs was not primarily influenced by their size due to the quantum confinement effect. Based on the analysis above, it can be suggested that high contents of pyridine-like nitrogen and low contents of oxygen may mainly induce the observed PL blue-shift of NGQDs.

## 4. Conclusions

In summary, NGQDs with high-content N were synthesized through the irradiation of GQDs in NH_3_ for different times. It was found that the fluorescence of GQDs could be significantly turned from pale yellow to blue-green, and a maximum blue-shift of approximately 47 nm could be obtained. Their superior optical properties should enable the use of NGQDs in numerous applications including multicolor light-emitting devices, biological applications, and photovoltaics.

## Figures and Tables

**Table 1:** The contents of nitrogen (N), oxygen (O), pyridine-like nitrogen (N-6), amino-like nitrogen (N-A), pyrrolic-like nitrogen (N-5), and graphitic-like nitrogen (N-Q) of the GQD and NGQD samples.

Samples (at %)	N-6	N-A	N-5	N-Q	N	O
GQDs	0	0	0	0	0	58.88
NGQDs-10	3.25	11.62	2.49	0.72	18.08	29.17
NGQDs-30	7.56	10.81	3.73	0.46	22.56	18.80
NGQDs-50	7.41	11.18	1.39	0.44	20.42	20.24
NGQDs-70	6.08	7.94	0.92	0.40	15.34	19.12
NGQDs-90	6.33	9.65	2.83	0.11	18.92	21.58

## Supplementary Materials

The following are available online at www.mdpi.com/1996-1944/10/11/1328/s1, Figure S1: (a) TEM image of rGQDs-70; (b) Diameter distribution of rGQDs-70, Figure S2: (a) Raman spectra and (b) FTIR spectra of GQDs, and rGQDs samples, Figure S3: XPS spectra of GQDs and the rGQDs samples obtained at different irradiation time, Figure S4: (a) Normalized PL Intensity of GQDs and rGQDs samples obtained at different irradiation time excited at 400 nm; (b) Optical photograph of the GQDs, rGQDs samples obtained at different irradiation time dispersed in water under 365 nm wavelength of irradiation.
