# Supplementary material for: Tuning the Photoluminescence of Graphene Quantum Dots by Photochemical Doping with Nitrogen
Source: Materials (Basel). 2017 Nov 20;10(11):1328. doi: 10.3390/ma10111328 (PMC5706275; doi:10.3390/ma10111328)
Supplement: Supplementary file 1 [file materials-10-01328-s001.pdf]

Supplementary Materials: Tuning the Photoluminescence of Graphene Quantum Dots by Photochemical Doping with Nitrogen

Xiaofen Xu, Fuhua Gao, Xiaohua Bai, Fuchi Liu, Wenjie Kong and Ming Li

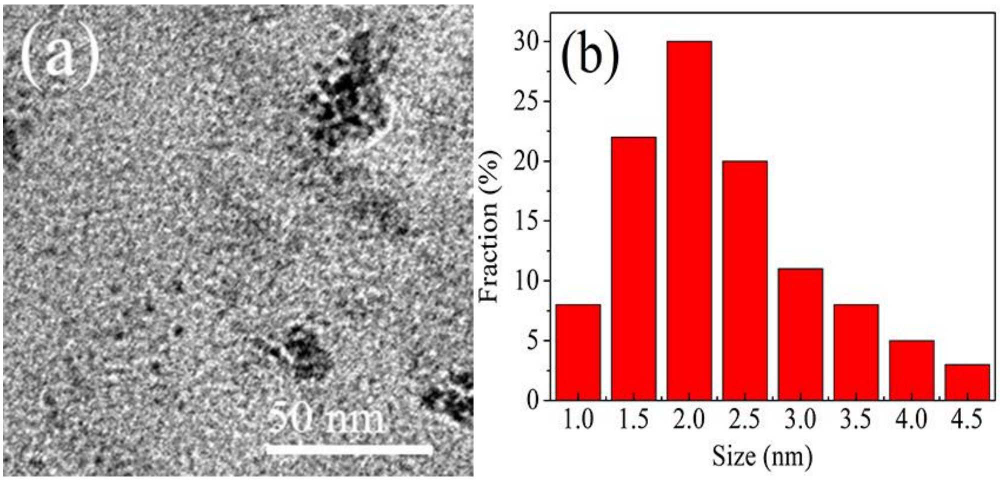

Figure S1: (**a**) TEM image of rGQDs-70; (**b**) Diameter distribution of rGQDs-70.

Figure S1 show the typical transmission electron microscopy (TEM) image and diameter distribution of rGQDs-70. It's found that rGQDs-70 relatively is uniform with diameters of about 1-4.5 nm. According to the calculations, one can found that the averaged diameter of rGQDs-70 is ca. 2.2 nm.

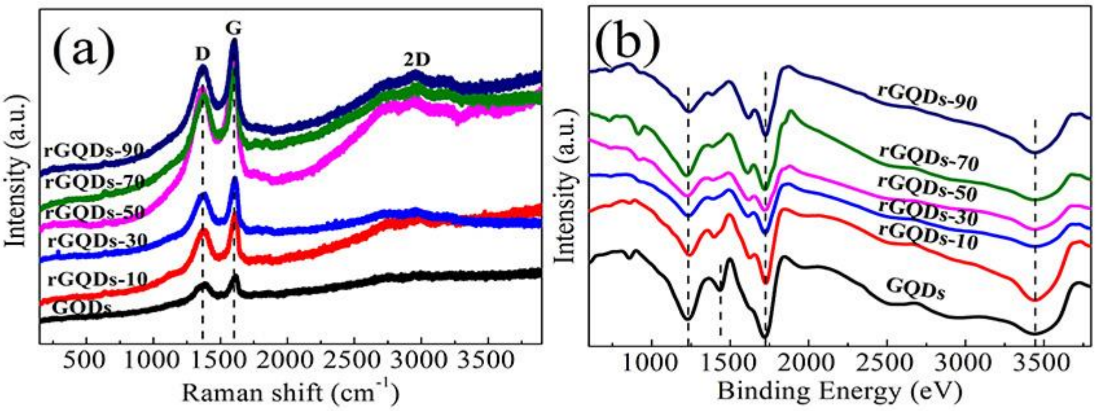

Figure S2: (**a**) Raman spectra and (**b**) FTIR spectra of GQDs, and rGQDs samples.

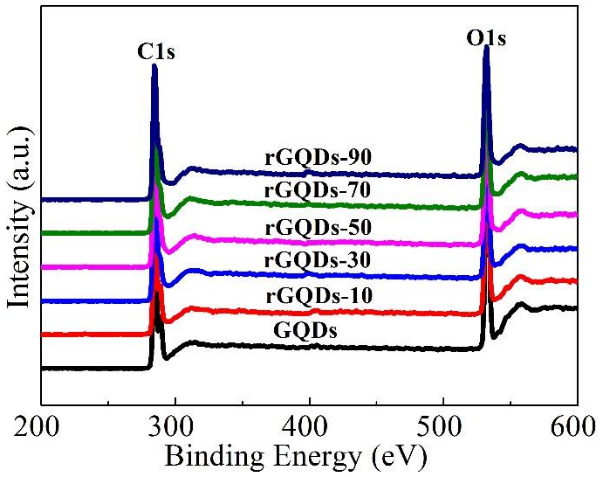

Figure S3: XPS spectra of GQDs and the rGQDs samples obtained at different irradiation time.

Figure S3 shows the typical XPS results of GQDs and the rGQDs samples. One can clearly see that compared to GQDs, the rGQDs samples show decrease in the peak intensities of the oxygen functional groups. It may be due to the fact that considerable amount of oxygen-containing functional groups of GQDs were removed during irradiating.[1]

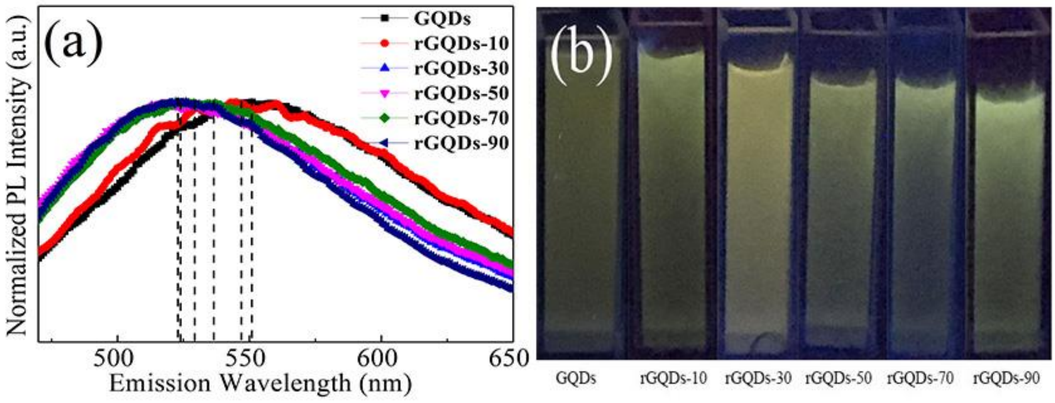

Figure S4: (**a**) Normalized PL Intensity of GQDs and rGQDs samples obtained at different irradiation time excited at 400 nm; (**b**) Optical photograph of the GQDs, rGQDs samples obtained at different irradiation time dispersed in water under 365 nm wavelength of irradiation.

Figure S4a shows the Normalized PL Intensity of GQDs and rGQDs samples obtained at different irradiation time excited at 400 nm. It is found that compared to that of GQDs, the PL of all the rGQDs samples exhibits a little blue-shift. The peak position of rGQDs is 545 nm for rGQDs-10, 535 nm for rGQDs-30, 518 nm for rGQDs-50, 523 nm for rGQDs-70, and 524 nm for rGQDs-90, respectively. Figure S4b shows the similar yellow luminescence of GQDs and rGQDs samples.

References

1. Dutta Chowdhury, A.;Doong, R.-A., Highly Sensitive and Selective Detection of Nanomolar Ferric Ions Using Dopamine Functionalized Graphene Quantum Dots. *ACS Appl. Mater. Interfaces* **2016**, *8*, 21002-10.

© 2017 by the authors. Submitted for possible open access publication under the terms and conditions of the Creative Commons Attribution (CC BY) license (http://creativecommons.org/licenses/by/4.0/).
